# Supplementary material for: Single and Joined Behaviour of Circulating Biomarkers and Metabolic Parameters in High-Fit and Low-Fit Healthy Females
Source: Int J Mol Sci. 2023 Feb 20;24(4):4202. doi: 10.3390/ijms24044202 (PMC9960642; doi:10.3390/ijms24044202)
Supplement: Supplementary file 1 [file Figure S3 Correlation plot Baseline.pdf › Figure S3 Correlation plot Baseline.pdf]

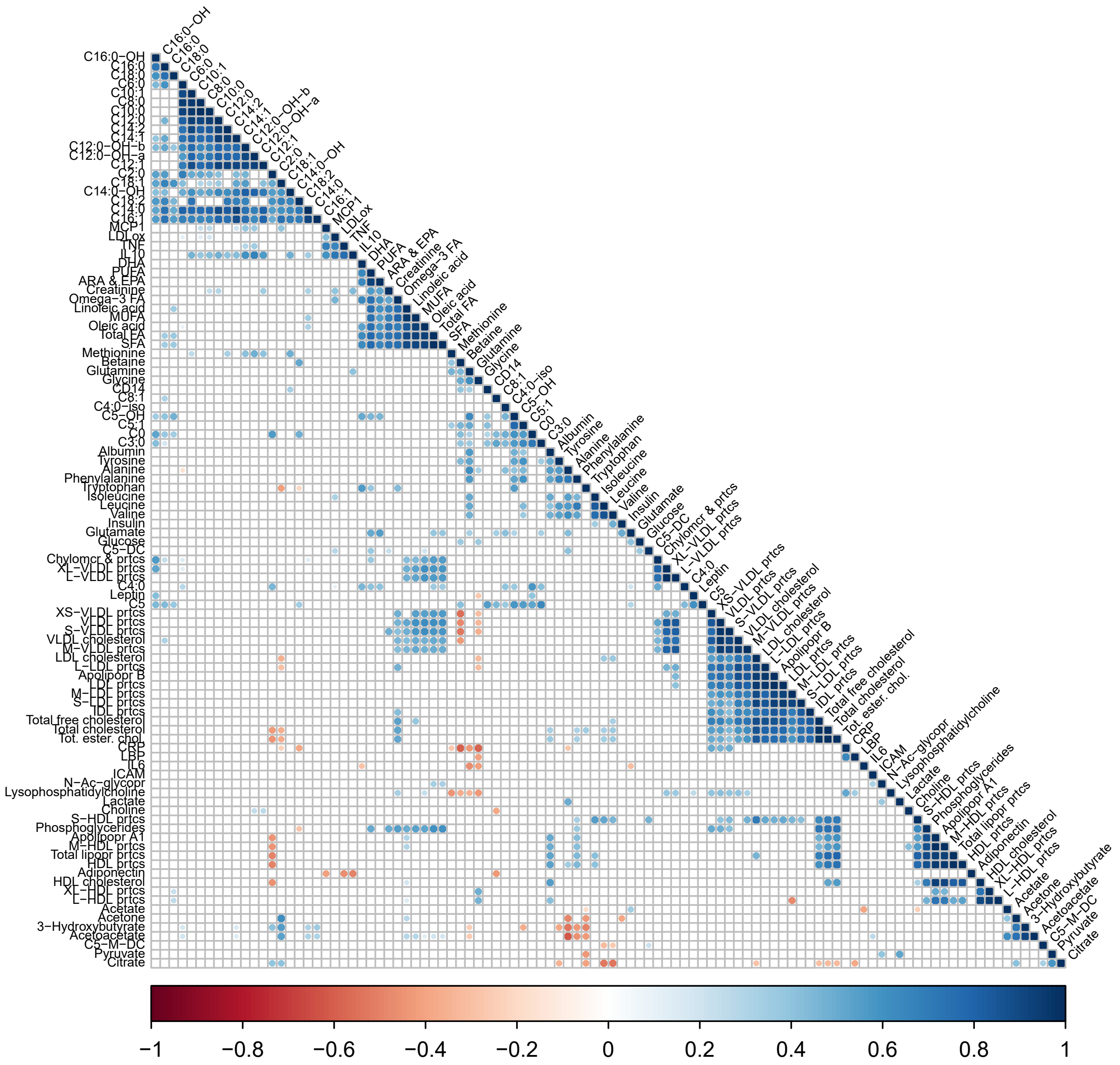

Supplementary Figure S 3: The correlation matrix based on Spearman correlation coefficients between biomarker and metabolic parameter pairs at baseline only. Spearman rank correlation analysis was performed on the scaled biomarker values for all biomarker pairs using the combined data of high-fit and low-fit females at baseline. Relationships were considered statistically significant when $P < 0.05$. Significant relationships are indicated in red (negative correlation) or blue (positive correlation). Non-significant relationships (P [illegible] 0.05) are left blank.
