# Supplementary material for: Single and Joined Behaviour of Circulating Biomarkers and Metabolic Parameters in High-Fit and Low-Fit Healthy Females
Source: Int J Mol Sci. 2023 Feb 20;24(4):4202. doi: 10.3390/ijms24044202 (PMC9960642; doi:10.3390/ijms24044202)
Supplement: Supplementary file 1 [file ijms-24-04202-s001.zip › Figure S1 Eurecat vs Nightingale.pdf]

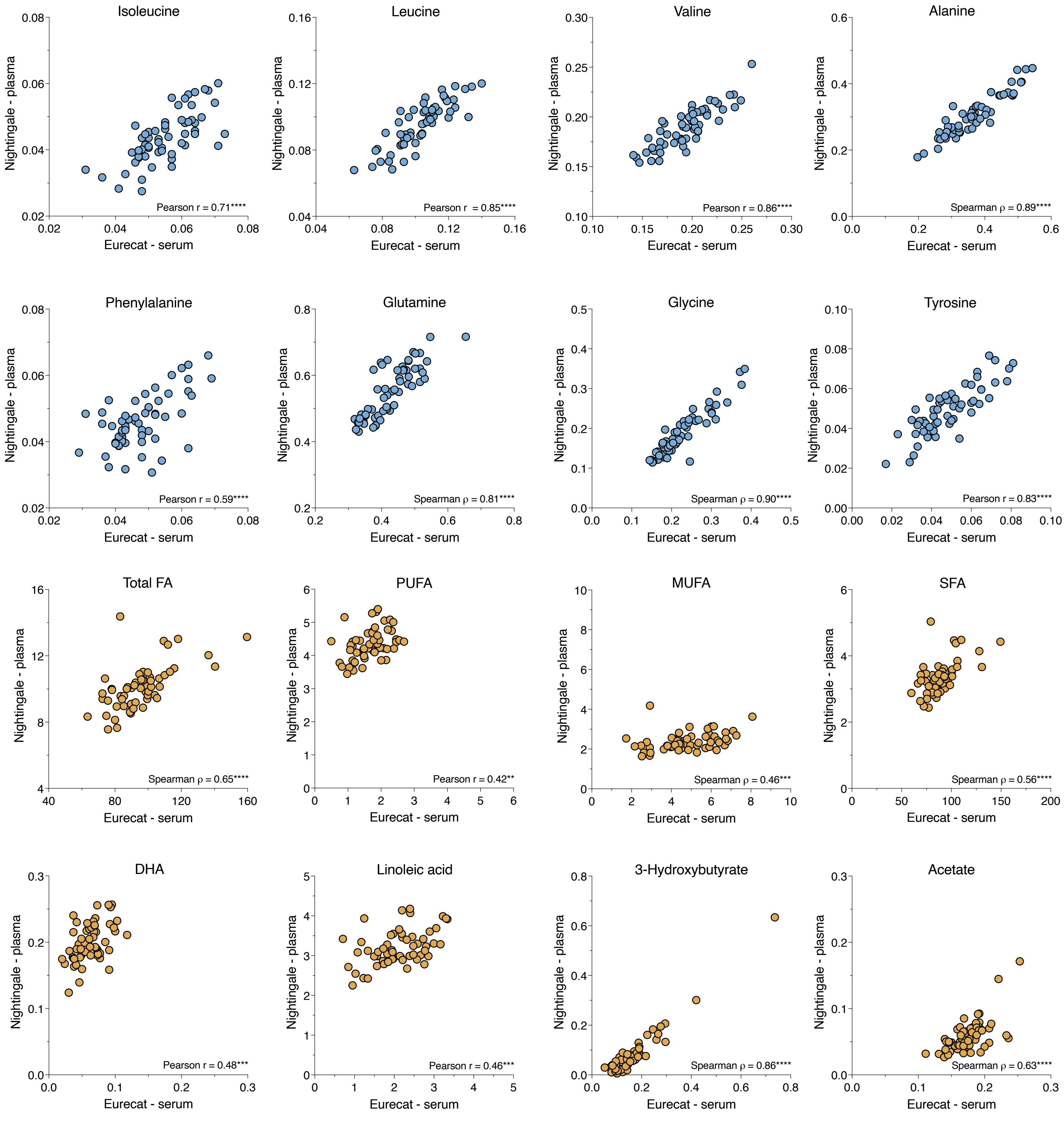

Supplementary Figure S1: Correlations between biomarkers and metabolic parameters in serum and plasma with two platforms. **(A - P)** Correlations of amino acid derivatives (A - H, blue) or fatty acids (I - P, beige) analyzed in serum at Eurecat (x-axis) or in plasma at Nightingale (y-axis). All concentrations are in mmol/L. Pearson correlation coefficients (r) were calculated normally distributed variables and Spearman rank correlation coefficients (ρ) were calculated for not-normally distributed variables. **** P < 0.0001, *** P < 0.001, ** P < 0.01.
