# Supplementary material for: Single and Joined Behaviour of Circulating Biomarkers and Metabolic Parameters in High-Fit and Low-Fit Healthy Females
Source: Int J Mol Sci. 2023 Feb 20;24(4):4202. doi: 10.3390/ijms24044202 (PMC9960642; doi:10.3390/ijms24044202)
Supplement: Supplementary file 1 [file ijms-24-04202-s001.zip › Figure S2 PCA.pdf]

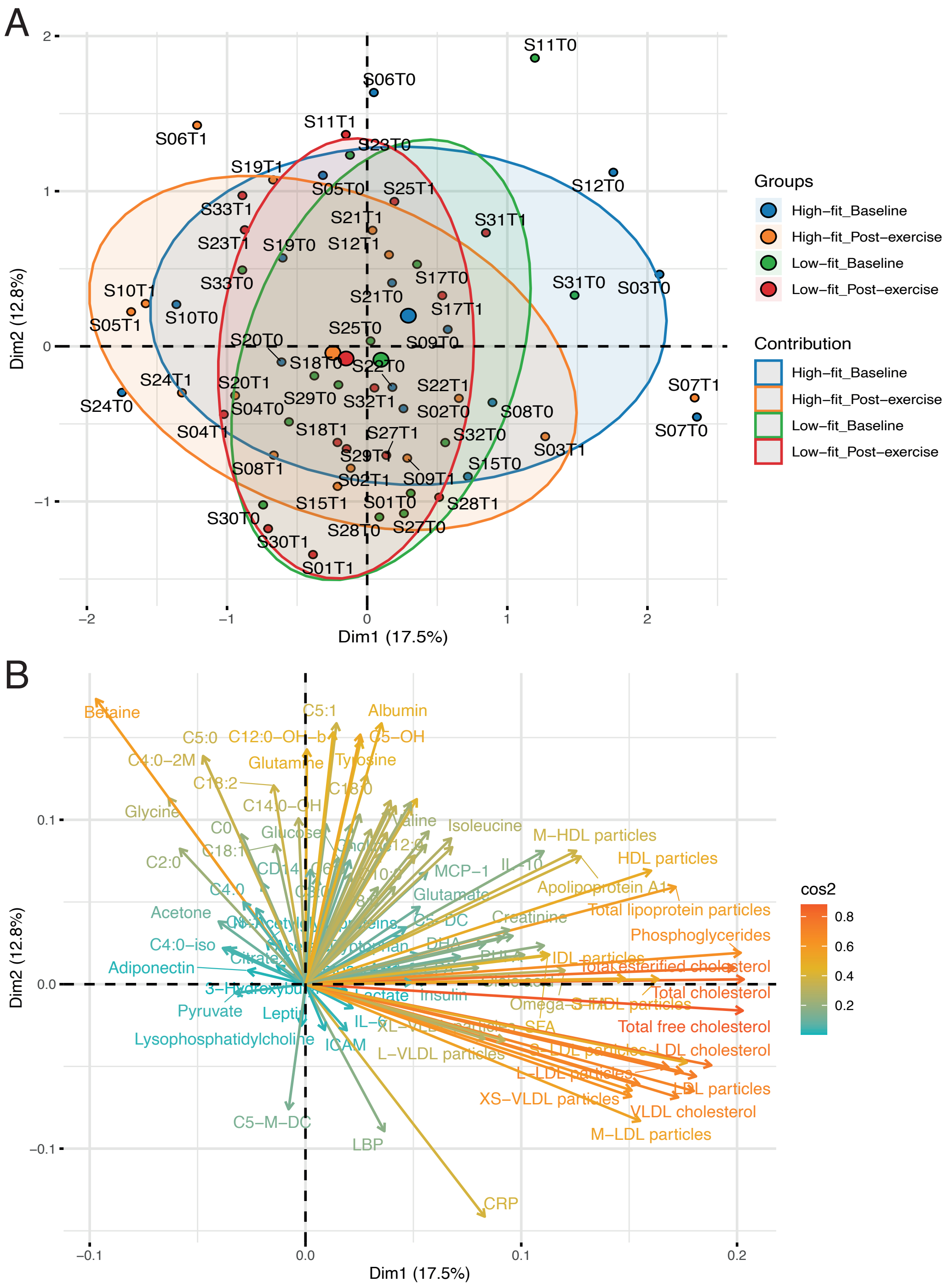

Supplementary Figure S2: Principal component analysis (PCA) of the biomarker and metabolic parameter profiles. (A) PCA scores plot showing the separation of high-fit females at baseline (blue) and post-exercise (orange) and low-fit females at baseline (green) and post-exercise (red) on PC1 and PC2. **(B)** PCA loadings plot showing the biomarker contribution (indicating by cos2) to PC1 and PC2. Small contributions are indicated in turquoise and large contributions are indicated in dark orange.
