# Supplementary material for: Single and Joined Behaviour of Circulating Biomarkers and Metabolic Parameters in High-Fit and Low-Fit Healthy Females
Source: Int J Mol Sci. 2023 Feb 20;24(4):4202. doi: 10.3390/ijms24044202 (PMC9960642; doi:10.3390/ijms24044202)
Supplement: Supplementary file 1 [file ijms-24-04202-s001.zip › Table S10.pdf]

Supplementary Table S10: Selected metabolites from Nightingale platform

Metabolite	Performed analyses*
Isoleucine	B
Leucine	B
Valine	B
Alanine	B
Glutamine	B
Glycine	B
Phenylalanine	B
Tyrosine	B
Glucose	A
Lactate	A
Pyruvate	A
Citrate	A
Total fatty acids (FAs)	B
Polyunsaturated fatty acids (PUFA)	B
Monounsaturated fatty acids (MUFA)	B
Saturated fatty acids (SFA)	B
Linoleic acid	B
Docosahexaenoic acid (DHA)	B
Phosphoglycerides	A
3-Hydroxybutyrate	A, B
Acetate	A, B
Acetoacetate	A
Acetone	A
Total cholesterol	A
VLDL cholesterol	A
LDL cholesterol	A
HDL cholesterol	A
Total esterified cholesterol	A
Total free cholesterol	A
Apolipoprotein B	A
Apolipoprotein A1	A
Total concentration of lipoprotein particles	A
Concentration of VLDL particles	A
Concentration of LDL particles	A
Concentration of HDL particles	A
Concentration of chylomicrons and extremely large VLDL particles	A
Concentration of very large VLDL particles	A
Concentration of large VLDL particles	A
Concentration of medium VLDL particles	A
Concentration of small VLDL particles	A
Concentration of very small VLDL particles	A
Concentration of IDL particles	A
Concentration of large LDL particles	A

Metabolite	Performed analyses*
Concentration of medium LDL particles	A
Concentration of small LDL particles	A
Concentration of very large HDL particles	A
Concentration of large HDL particles	A
Concentration of medium HDL particles	A
Concentration of small HDL particles	A
Creatinine	A
Albumin	A

*A = used for main analyses (RM-ANOVA, main effects, PCA, heatmaps, correlation matrices), B = used for correlation analysis (comparison with serum levels in Eurecat platform)
