# Supplementary material for: Single and Joined Behaviour of Circulating Biomarkers and Metabolic Parameters in High-Fit and Low-Fit Healthy Females
Source: Int J Mol Sci. 2023 Feb 20;24(4):4202. doi: 10.3390/ijms24044202 (PMC9960642; doi:10.3390/ijms24044202)
Supplement: Supplementary file 1 [file ijms-24-04202-s001.zip › Table S11.pdf]

Supplementary Table S11: Standards and internal standards used for LC-MS/MS analyses

Standards	Company[#]	Catalogue number #
L-carnitine (C0)	CIL	NSK-B-US-1
O-acetyl-L-carnitine (C2:0)	CIL	NSK-B-US-1
O-propionyl-L-carnitine (C3:0)	CIL	NSK-B-US-1
O-butyryl-L-carnitine (C4:0)	CIL	NSK-B-US-1
O-isovaleryl-L-carnitine (C5:0)	CIL	NSK-B-US-1
O-glutaryl-L-carnitine (C5:0-DC)	CIL	NSK-B-G1-US-1
O-3-hydroxyisolaveryl-L-carnitine (C5:0-OH	CIL	NSK-B-G1-US-1
O-octanoyl-L-carnitine (C8:0)	CIL	NSK-B-US-1
O-dodecanoyl-L-carnitine (C12:0)	CIL	NSK-B-G1-US-1
O-myristoyl-L-carnitine (C14:0)	CIL	NSK-B-US-1
O-palmitoyl-L-carnitine (C16:0)	CIL	NSK-B-US-1
O-2-DL-hydroxypalmitoyl-L-carnitine (C16:0-OH)	CIL	NSK-B-G1-US-1
O-octadecanoyl-L-carnitine (C18:0)	CIL	NSK-B-G1-US-1
**Internal standards**	**Company[#]**	**Catalogue number #**
L-carnitine (C0)	CIL	NSK-B-1
O-acetyl-L-carnitine (C2:0)	CIL	NSK-B-1
O-propionyl-L-carnitine (C3:0)	CIL	NSK-B-1
O-butyryl-L-carnitine (C4:0)	CIL	NSK-B-1
O-isovaleryl-L-carnitine (C5:0)	CIL	NSK-B-1
O-octanoyl-L-carnitine (C8:0)	CIL	NSK-B-1
O-myristoyl-L-carnitine (C14:0)	CIL	NSK-B-1
O-palmitoyl-L-carnitine (C16:0)	CIL	NSK-B-1

[#]CIL = Cambridge Isotope Laboratories
