# Supplementary material for: Single and Joined Behaviour of Circulating Biomarkers and Metabolic Parameters in High-Fit and Low-Fit Healthy Females
Source: Int J Mol Sci. 2023 Feb 20;24(4):4202. doi: 10.3390/ijms24044202 (PMC9960642; doi:10.3390/ijms24044202)
Supplement: Supplementary file 1 [file ijms-24-04202-s001.zip › Table S8 (Corr P values).pdf]

Supplementary Table S8: Highly correlated biomarkers

Biomarker	TNF.a	MCP.1
TNFa		1.0E-11
MCP1	1.0E-11	

Biomarker	CRP	Glycine
CRP		2.2E-10
Glycine	2.2E-10	

Biomarker	Isoleucine	Leucine	Valine
Isoleucine		8.9E-16	8.8E-12
Leucine	8.9E-16		<1E-16
Valine	8.8E-12	<1E-16	

Biomarker	I	II	III	IV	V	VI	VII	VIII
SFA (I)		<1E-16	2.6E-09					
Total.FA (II)	<1E-16		3.8E-11		6.7E-09	2.5E-09	4.4E-09	
Omega3.FA (III)	2.6E-09	3.8E-11		1.5E-11	4.6E-14	6.2E-13	1.0E-11	
Linoleic.acid (IV)			1.5E-11		<1E-16	<1E-16	1.4E-12	
MUFA (V)		6.7E-09	4.6E-14	<1E-16		<1E-16	2.3E-13	
Oleic.acid (VI)		2.5E-09	6.2E-13	<1E-16	<1E-16		4.9E-15	3.9E-09
PUFA (VII)		4.4E-09	1.0E-11	1.4E-12	2.3E-13	4.9E-15		<1E-16
ARA...EPA (VIII)						3.9E-09	<1E-16	

Biomarker	i	ii	ii
3-Hydroxybutyrate (i)		<1E-16	1.5E-08
Acetoacetate (ii)	<1E-16		3.9E-10
Acetone (iii)	1.5E-08	3.9E-10	

Biomarker	C0	C3.0	C4.0.2M	C5.0	C5.OH	C5.1
C0		1.2E-11				
C3.0	1.2E-11		5.2E-09	5.2E-09	1.3E-09	
C4.0.2M		5.2E-09		<1E-16	7.8E-10	
C5.0		5.2E-09	<1E-16		7.8E-10	
C5.OH			7.8E-10	7.8E-10		3.3E-12
C5.1					3.3E-12	

Biomarker	C6.0	C8.0	C10.0	C10.1	C14.2	C14.1	C14.0	C12.0	C12.1	C12.0.OH.a	C12.0.OH.b	C14.0.OH	C16.1	C16.0	C18.1	C18.2	C16.0.OH	C18.0
C6.0		4.2E-12	7.1E-11			6.7E-09												
C8.0	4.2E-12		7.1E-11	2.6E-14	7.9E-11	4.4E-15	1.3E-09	<1E-16	8.7E-12									
C10.0	7.1E-11	<1E-16		1.2E-14	3.3E-12	<1E-16	1.4E-10	<1E-16	1.0E-14	8.9E-09			7.3E-09					
C10.1		2.6E-14	1.2E-14		<1E-16	2.0E-12	2.9E-09	1.5E-14	1.8E-14	7.9E-13	6.5E-09							
C14.2		7.9E-11	3.3E-12	<1E-16		<1E-16	8.5E-12	<1E-16	<1E-16	1.6E-14			4.6E-09					
C14.1	6.7E-09	4.4E-15	<1E-16	2.0E-12	<1E-16		<1E-16	<1E-16	<1E-16	4.2E-10	1.8E-11	3.3E-10	<1E-16		4.1E-09			
C14.0		1.3E-09	1.4E-10	2.9E-09	8.5E-12	<1E-16		<1E-16	1.0E-14				<1E-16	1.4E-11				
C12.0		<1E-16	<1E-16	1.5E-14	<1E-16	<1E-16	<1E-16		<1E-16	1.4E-12	1.1E-10		2.7E-13					
C12.1		8.7E-12	1.0E-14	1.8E-14	<1E-16	<1E-16	1.0E-14	<1E-16		4.0E-15	1.1E-15	8.4E-11	7.5E-14					
C12.0.OH.a			8.9E-09	7.9E-13	1.6E-14	4.2E-10		1.4E-12	4.0E-15		2.4E-10	4.8E-11						
C12.0.OH.b				6.5E-09		1.8E-11		1.1E-10	1.1E-15	2.4E-10		1.7E-14	2.1E-10		2.1E-09			
C14.0.OH						3.3E-10			8.4E-11	4.8E-11	1.7E-14				7.7E-09			
C16.1					4.6E-09	<1E-16	<1E-16	2.7E-13	7.5E-14		2.1E-10			6.2E-10	1.4E-12			
C18.2															2.8E-10			
C18.1						4.1E-09							1.4E-12			2.8E-10		
C16.0							1.4E-11						6.2E-10				1.2E-10	3.9E-10
C16.0.OH														1.2E-10				
C18.0														3.9E-10				

Biomarker	A	B	C	D	E	F	G	H	I	J	K	L	M	N
Total.cholesterol (A)		<1E-16	<1E-16	<1E-16	1.8E-12	1.3E-11	5.0E-11	2.5E-14	2.7E-12					
Total.esterified.cholesterol (B)	<1E-16		<1E-16	1.3E-15	6.4E-11	2.7E-10	1.1E-09	2.5E-13	1.1E-11					
Total.free.cholesterol (C)	<1E-16	<1E-16		<1E-16	2.2E-16	9.8E-15	1.5E-14	<1E-16	4.1E-13	6.7E-09	1.2E-09	3.2E-10	9.6E-10	
LDL.cholesterol (D)	<1E-16	1.3E-15	<1E-16		<1E-16	<1E-16	<1E-16	1.9E-10	<1E-16	<1E-16	1.2E-10		3.4E-12	
Apolipoprotein.B (E)	1.8E-12	6.4E-11	2.2E-16	<1E-16		<1E-16	<1E-16	3.1E-15	<1E-16	<1E-16	<1E-16	1.8E-12	<1E-16	1.9E-12
LDL.particles (F)	1.3E-11	2.7E-10	9.8E-15	<1E-16	<1E-16		<1E-16	6.0E-13	<1E-16	<1E-16	2.2E-16	1.1E-10	5.6E-15	1.2E-10
L.LDL.particles (G)	5.0E-11	1.1E-09	1.5E-14	<1E-16	<1E-16	<1E-16		5.1E-13	<1E-16	4.0E-14	<1E-16	2.3E-12	6.7E-16	1.4E-11
IDL.particles (H)	2.5E-14	2.5E-13	<1E-16	1.9E-10	3.1E-15	6.0E-13	5.1E-13		3.5E-14			1.5E-09		
S.LDL.particles (I)	2.7E-12	1.1E-11	4.1E-13	<1E-16	<1E-16	<1E-16	<1E-16	3.5E-14		<1E-16			2.1E-09	
M.LDL.particles (J)			6.7E-09	<1E-16	<1E-16	<1E-16	4.0E-14		<1E-16		7.8E-10		8.7E-11	
VLDL.cholesterol (K)			1.2E-09	1.2E-10	<1E-16	2.2E-16	<1E-16			7.8E-10		<1E-16	<1E-16	<1E-16
XS.VLDL.particles (L)			3.2E-10		1.8E-12	1.1E-10	2.3E-12	1.5E-09			<1E-16		2.0E-09	8.9E-16
M.VLDL.particles (M)			9.6E-10	3.4E-12	<1E-16	5.6E-15	6.7E-16		2.1E-09	8.7E-11	<1E-16	2.0E-09		<1E-16
VLDL.particles (N)					1.9E-12	1.2E-10	1.4E-11				<1E-16	8.9E-16	<1E-16	
S.VLDL.particles (O)											<1E-16	4.7E-10	<1E-16	<1E-16
XL.VLDL.particles (P)													3.9E-10	7.4E-12
L.VLDL.particles (Q)											7.7E-09		3.4E-13	3.1E-14
Chylomicrons...XXL.VLDL.particles (R)														
Total.lipoprotein.particles (S)	7.0E-11	9.2E-12												
Phosphoglycerides (T)	7.2E-11	5.1E-11	4.5E-10											
Apolipoprotein.A1 (U)														
HDL.particles (V)	9.8E-09	1.6E-09												
M.HDL.particles (W)														
HDL.cholesterol (X)														
S.HDL.particles (Y)														
L.HDL.particles (Z)														
XL.HDL.particles (AB)														

Biomarker	O	P	Q	R	S	T	U	V	W	X	Y	Z	AB
Total.cholesterol (A)					7.0E-11	7.2E-11		9.8E-09					
Total.esterified.cholesterol (B)					9.2E-12	5.1E-11		1.6E-09					
Total.free.cholesterol (C)						4.5E-10							
LDL.cholesterol (D)													
Apolipoprotein.B (E)													
LDL.particles (F)													
L.LDL.particles (G)													
IDL.particles (H)													
S.LDL.particles (I)													
M.LDL.particles (J)													
VLDL.cholesterol (K)	<1E-16		7.7E-09										
XS.VLDL.particles (L)	4.7E-10												
M.VLDL.particles (M)	<1E-16	3.9E-10	3.4E-13										
VLDL.particles (N)	<1E-16	7.4E-12	3.1E-14										
S.VLDL.particles (O)		3.8E-14	<1E-16										
XL.VLDL.particles (P)	3.8E-14		<1E-16	3.7E-12									
L.VLDL.particles (Q)	<1E-16	<1E-16											
Chylomicrons...XXL.VLDL.particles (R)		3.7E-12											
Total.lipoprotein.particles (S)						<1E-16	<1E-16	<1E-16	<1E-16	<1E-16	4.1E-13		
Phosphoglycerides (T)					<1E-16		<1E-16	<1E-16	<1E-16	1.2E-12	2.3E-09		
Apolipoprotein.A1 (U)					<1E-16	<1E-16		<1E-16	<1E-16	<1E-16		1.3E-09	
HDL.particles (V)					<1E-16	<1E-16	<1E-16		<1E-16	<1E-16	1.0E-11		
M.HDL.particles (W)					<1E-16	<1E-16	<1E-16	<1E-16		<1E-16		8.3E-09	
HDL.cholesterol (X)					<1E-16	1.2E-12	<1E-16	<1E-16	<1E-16			4.4E-16	
S.HDL.particles (Y)					4.1E-13			1.0E-11					
L.HDL.particles (Z)							1.3E-09		8.3E-09	4.4E-16			<1E-16
XL.HDL.particles (AB)												<1E-16	

Biomarker interactions were selected using unsupervised correlation analysis with P<1E-9. All independent sets at this cutoff are indicated. All correlations in the immediate vicinity of these sets at P<1E-8 are indicated for improved interpretation.
Of note, correlation P values were of rapidly decreasing significance outside these sets. A few additional independent sets could be observed at lower, but still highly significant, cut-offs (e.g. P<1E-7). These are not given, to emphasize the most robust sets.
