# Supplementary material for: Single and Joined Behaviour of Circulating Biomarkers and Metabolic Parameters in High-Fit and Low-Fit Healthy Females
Source: Int J Mol Sci. 2023 Feb 20;24(4):4202. doi: 10.3390/ijms24044202 (PMC9960642; doi:10.3390/ijms24044202)
Supplement: Supplementary file 1 [file ijms-24-04202-s001.zip › Table S9.pdf]

**Supplementary Table S9:** ELISA kits used for serum and plasma biomarker and metabolic parameter analysis

| Biomarker | Matrix | Company | Catalogue number |
|---|---|---|---|
| Leptin | Serum | R&D Systems[a] | DLP00 |
| Insulin | Serum | Mercodia[b] | 10-1113-01 |
| Adiponectin | Serum | R&D Systems | DRP300 |
| TNF | Plasma | R&D Systems | HSTA00E |
| IL6 | Plasma | R&D Systems | HS600C |
| IL10 | Plasma | Invitrogen[c] | BMS215HS |
| CRP | Plasma | R&D Systems | DCRP00 |
| CD14 | Plasma | R&D Systems | DC140 |
| MCP1 | Plasma | R&D Systems | DCP00 |
| Soluble ICAM-1 | Plasma | R&D Systems | BBE1B |
| LBP | Plasma | Hycult Biotech[d] | HK315-01 |
| Oxidized LDL | Plasma | Mercodia | 10-1143-01 |

[a]R&D systems Inc., Minneapolis, MN, Canada
[b]Mercodia, Uppsala, Sweden
[c]Invitrogen, Thermo Fisher Scientific, Inc., Waltham, MA, USA
[d]Hycult Biotech, Uden, Netherlands
