# Supplementary material for: Single and Joined Behaviour of Circulating Biomarkers and Metabolic Parameters in High-Fit and Low-Fit Healthy Females
Source: Int J Mol Sci. 2023 Feb 20;24(4):4202. doi: 10.3390/ijms24044202 (PMC9960642; doi:10.3390/ijms24044202)
Supplement: Supplementary file 1 [file ijms-24-04202-s001.zip › Tables S1,2,3.pdf]

Supplementary Table S1: Peptide hormone markers

Biomarker[a]	Matrix	Method	Concentration	Low-fit (Baseline)	Low-fit (Post-exercise)	High-fit (Baseline)	High-fit (Post-exercise)	Transformation[#]	Fitness level effect			Recent exercise effect			Interaction effect		
									P_{group} raw	P_{group} adj	Partial η^2	$P_{exercise}$ raw	$P_{exercise}$ adj	Partial η^2	$P_{group*exercise}$ raw	$P_{group*exercise}$ adj	Partial η^2
Leptin	Serum	ELISA	ng/mL	12.8 [7.1 – 18.1]	16.8 [8.8 – 24.5]	5.6 [3.9 – 6.6]	4.4 [3.7 – 5.6]	Log	**0.001**	**0.076**	0.339	0.391	0.570	0.026	0.067	0.346	0.114
Insulin	Serum	ELISA	mU/L	4.1 [3.4 – 5.0]	3.9 [2.8 – 4.8]	3.3 [2.6 – 4.3]	2.5 [2.2 – 3.7]	Log	**0.039**	0.464	0.144	**0.037**	0.105	0.148	0.439	0.656	0.022
Adiponectin	Serum	ELISA	µg/mL	24.0 [16.0 – 29.6]	28.6 [21.3 – 33.6]	19.4 [13.9 – 27.4]	32.9 [27.2 – 38.9]	Log	0.914	0.931	4.29E-04	**1.55E-04**	**0.001**	0.405	0.143	0.409	0.075

Data is represented as mean [IQR]. Normally distributed data is represented as mean (SD) and non-normally distributed data is represented as mean [IQR]. Raw P-values were adjusted using an FDR of 10%. Significant raw P-values are indicated in **bold**. Significant FDR-adjusted P-values (< 0.10) are indicated in **underlined bold**.

[#]Log-transformation was applied on not-normally distributed variables.

[a] Analyzed using the EURECAT platform.

Supplementary Table S2: Inflammation and oxidative stress related markers

Biomarker[a]	Matrix	Method	Concentration	Low-fit (Baseline)	Low-fit (Post-exercise)	High-fit (Baseline)	High-fit (Post-exercise)	Transformation[#]	Fitness level effect			Recent exercise effect			Interaction effect		
									P_{group} raw	P_{group} adj	Partial η^2	$P_{exercise}$ raw	$P_{exercise}$ adj	Partial η^2	$P_{group*exercise}$ raw	$P_{group*exercise}$ adj	Partial η^2
TNF	Plasma	ELISA	pg/mL	0.44 [0.38 – 0.58]	0.39 [0.36 – 0.43]	0.60 [0.49 – 0.78]	0.34 [0.28 – 0.39]	Log	0.693	0.897	0.006	**1.20E-05**	**3.09E-04**	0.502	**0.009**	0.247	0.220
IL6	Plasma	ELISA	pg/mL	1.02 [0.64 – 1.29]	0.66 [0.41 – 1.16]	0.76 [0.55 – 1.41]	0.43 [0.26 – 0.58]	Log	0.154	0.640	0.071	**0.004**	**0.017**	0.256	**0.036**	0.325	0.147
IL10	Plasma	ELISA	pg/mL	0.57 [0.31 – 8.61]	0.33 [0.09 – 0.60]	7.93 [4.82 – 11.2]	0.43 [0.21 – 0.70]	Log	**0.025**	0.464	0.167	**3.46E-04**	**0.003**	0.372	0.116	0.372	0.086
CRP	Serum	ELISA	µg/mL	0.66 [0.51 – 1.78]	0.85 [0.63 – 2.27]	0.31 [0.14 – 1.91]	0.43 [0.22 – 2.17]	Log	0.065	0.606	0.117	**3.31E-04**	**0.003**	0.374	0.250	0.547	0.047
Soluble CD14	Plasma	ELISA	µg/mL	1.26 [1.15 – 1.43]	1.38 [1.23 – 1.46]	1.33 [1.15 – 1.43]	1.56 [1.26 – 1.68]	Log	0.546	0.897	0.013	**0.004**	**0.015**	0.265	0.082	0.352	0.104
MCP1	Plasma	ELISA	pg/mL	166 [144 – 267]	131 [111 – 144]	244 [212 – 280]	100 [89.1 – 111]	Inverse	0.379	0.855	0.028	**1.21E-07**	**4.16E-06**	0.638	**0.005**	0.247	0.247
Soluble ICAM1	Plasma	ELISA	ng/mL	161 [117 – 184]	151 [129 – 189]	169 [165 – 182]	189 [129 – 197]	Square	0.160	0.640	0.069	0.939	0.972	2.14×10^{-4}	0.709	0.815	0.005
LBP	Plasma	ELISA	µg/µL	6.84 [5.82 – 8.89]	6.62 [5.66 – 10.3]	6.35 [5.49 – 8.37]	6.21 [5.84 – 7.72]	Log	0.604	0.897	0.010	0.620	0.718	0.009	0.967	0.989	6.20E-05
N-acetyl glycoproteins	Serum	NMR	mmol/L	0.07 [0.06 – 0.08]	0.11 [0.10 – 0.13]	0.08 [0.06 – 0.09]	0.12 [0.11 – 0.13]	Log	0.513	0.897	0.015	**1.02E-07**	**4.16E-06**	0.642	0.938	0.982	2.24E-04
Oxidized LDL	Plasma	ELISA	U/L	103 [86 – 151]	85 [66 – 120]	190 [27 – 389]	82 [65 – 123]	Log	0.679	0.897	0.006	0.315	0.508	0.036	0.581	0.739	0.011

Data is represented as mean [IQR]. Normally distributed data is represented as mean (SD) and non-normally distributed data is represented as mean [IQR]. Raw P-values were adjusted using an FDR of 10%. Significant raw P-values are indicated in **bold**. Significant FDR-adjusted P-values (< 0.10) are indicated in **underlined bold**.

[#]Transformations (log, inverse, square) were applied on not-normally distributed variables.

[a] Analyzed using the EURECAT platform.

Supplementary Table S3: Metabolic markers

Metabolism class	Biomarker[a]	Matrix	Method	Concentration	Low-fit (Baseline)	Low-fit (Post-exercise)	High-fit (Baseline)	High-fit (Post-exercise)	Transformation[#]	Fitness level effect			Recent exercise effect			Interaction effect		
										P_{group} raw	P_{group} adj	Partial η^2	$P_{exercise}$ raw	$P_{exercise}$ adj	Partial η^2	$P_{group*exercise}$ raw	$P_{group*exercise}$ adj	Partial η^2
Protein and amino acid related metabolites[a]	Isoleucine	Serum	NMR	mmol/L	0.05 (0.01)	0.054 (0.010)	0.054 (0.006)	0.058 (0.009)	-	0.287	0.806	0.042	0.447	0.622	0.022	0.447	0.657	0.022
	Leucine	Serum	NMR	mmol/L	0.10 (0.01)	0.099 (0.014)	0.097 (0.011)	0.109 (0.019)	-	0.081	0.640	0.108	0.298	0.508	0.040	0.812	0.889	0.002
	Valine	Serum	NMR	mmol/L	0.20 (0.03)	0.204 (0.028)	0.185 (0.017)	0.202 (0.030)	-	0.991	0.931	0.000	**0.002**	**0.009**	0.316	0.764	0.855	0.003
	Alanine	Serum	NMR	mmol/L	0.36 (0.06)	0.355 (0.057)	0.341 (0.073)	0.402 (0.085)	-	0.187	0.664	0.064	0.060	0.146	0.125	0.385	0.615	0.028
	Phenylalanine	Serum	NMR	mmol/L	0.04 (0.01)	0.044 (0.005)	0.047 (0.007)	0.051 (0.010)	-	0.129	0.640	0.083	0.927	0.972	3.18E-04	0.054	0.346	0.131
	Glutamine	Serum	NMR	mmol/L	0.42 [0.40 – 0.45]	0.422 [0.398 – 0.454]	0.389 [0.348 – 0.473]	0.465 [0.397 – 0.516]	Log	0.406	0.855	0.026	**0.005**	**0.018**	0.257	0.720	0.815	0.005
	Glutamate	Serum	NMR	mmol/L	0.07 [0.06 – 0.08]	0.071 [0.064 – 0.082]	0.080 [0.065 – 0.095]	0.065 [0.060 – 0.089]	Log	0.315	0.812	0.037	0.984	0.972	1.40E-05	0.170	0.443	0.069
	Glycine	Serum	NMR	mmol/L	0.20 [0.18 – 0.24]	0.204 [0.182 – 0.238]	0.218 [0.186 – 0.229]	0.233 [0.200 – 0.306]	Log	0.148	0.640	0.076	0.566	0.685	0.012	0.371	0.615	0.030
	Methionine	Serum	NMR	mmol/L	0.017 (0.007)	0.017 (0.007)	0.021 (0.014)	0.032 (0.014)	-	**0.010**	0.376	0.221	0.963	0.972	8.10E-05	0.188	0.450	0.063
	Tyrosine	Serum	NMR	mmol/L	0.047 (0.014)	0.047 (0.014)	0.048 (0.014)	0.051 (0.016)	-	0.676	0.897	0.007	0.549	0.673	0.013	0.320	0.615	0.037
	Tryptophan	Serum	NMR	mmol/L	0.057 (0.013)	0.057 (0.013)	0.051 (0.017)	0.053 (0.019)	-	0.592	0.897	0.011	0.270	0.472	0.045	0.685	0.812	0.006
	Betaine	Serum	NMR	mmol/L	0.03 [0.02 – 0.04]	0.031 [0.022 – 0.036]	0.027 [0.019 – 0.033]	0.032 [0.022 – 0.041]	Inverse Sqrt	0.740	0.897	0.004	**0.002**	**0.011**	0.293	0.477	0.680	0.019
Carbohydrate and TCA cycle metabolites[b]	Glucose	Plasma	NMR	mmol/L	4.80 (0.28)	4.78 (0.32)	4.85 (0.22)	4.81 (0.10)	-	0.620	0.897	0.009	0.363	0.559	0.030	0.710	0.815	0.005
	Lactate	Plasma	NMR	mmol/L	0.72 (0.16)	0.71 (0.192)	0.79 (0.17)	0.75 (0.23)	-	0.382	0.855	0.027	0.589	0.698	0.011	0.809	0.889	0.002
	Pyruvate	Plasma	NMR	mmol/L	0.054 (0.014)	0.056 (0.009)	0.054 (0.010)	0.061 (0.015)	-	0.531	0.897	0.014	0.123	0.257	0.083	0.343	0.615	0.032
	Citrate	Plasma	NMR	mmol/L	0.066 (0.009)	0.065 (0.013)	0.067 (0.010)	0.073 (0.012)	-	0.204	0.677	0.057	0.238	0.435	0.049	0.143	0.409	0.075
Lipid metabolites – Fatty acids[a, $]	Total FA chains	Serum	NMR	mmol/L	99 [92 – 105]	94 [90 – 100]	91 [83 – 110]	84 [76 – 98]	Log	0.310	0.812	0.038	**0.040**	0.111	0.147	0.344	0.615	0.033
	PUFA	Serum	NMR	mmol/L	1.63 (0.47)	2.00 (0.44)	1.58 (0.47)	1.57 (0.48)	-	0.127	0.640	0.084	0.074	0.169	0.114	0.056	0.346	0.129
	Omega-3 FA	Serum	NMR	mmol/L	0.167 (0.021)	0.179 (0.030)	0.161 (0.043)	0.160 (0.037)	-	0.197	0.677	0.061	0.515	0.655	0.016	0.475	0.680	0.019
	ARA & EPA	Serum	NMR	mmol/L	0.450 (0.085)	0.514 (0.086)	0.439 (0.101)	0.442 (0.086)	-	0.158	0.640	0.072	0.097	0.208	0.099	0.122	0.380	0.086
	DHA	Serum	NMR	mmol/L	0.074 (0.020)	0.065 (0.018)	0.060 (0.022)	0.051 (0.019)	-	**0.041**	0.464	0.146	**0.032**	**0.095**	0.159	0.987	0.989	$1.10x10^{-5}$
	Linoleic acid	Serum	NMR	mmol/L	1.88 (0.44)	2.57 (0.47)	1.94 (0.63)	2.00 (0.74)	-	0.165	0.640	0.070	**0.012**	**0.041**	0.213	**0.031**	0.325	0.162
	MUFA	Serum	NMR	mmol/L	4.28 (0.95)	5.72 (1.03)	4.52 (1.50)	4.62 (1.57)	-	0.276	0.806	0.044	**0.023**	**0.070**	0.177	**0.046**	0.336	0.140
	Oleic acid	Serum	NMR	mmol/L	1.63 [1.41 – 1.75]	2.03 [1.89 – 2.32]	1.60 [1.27 – 1.90]	1.74 [1.22 – 2.12]	Log	0.426	0.878	0.024	0.053	0.134	0.132	0.076	0.352	0.112
	SFA	Serum	NMR	mmol/L	93 [87 – 98]	86 [82 – 91]	86 [79 – 103]	79 [71 – 91]	Log	0.351	0.855	0.032	**0.012**	**0.041**	0.213	0.434	0.656	0.023
	Phosphoglycerides[b]	Plasma	NMR	mmol/L	2.15 [1.92 – 2.32]	2.02 [1.84 – 2.26]	2.15 [1.99 – 2.40]	2.11 [1.87 – 2.22]	Log	0.814	0.897	0.002	**2.24E-04**	**0.002**	0.390	0.614	0.772	0.009
	Acetate[b]	Plasma	NMR	mmol/L	0.046 [0.039 – 0.074]	0.068 [0.052 – 0.072]	0.050 [0.045 – 0.057]	0.051 [0.043 – 0.062]	Log	0.166	0.640	0.067	0.160	0.318	0.069	0.294	0.613	0.039
Lipid metabolites – Cholines[b]	Choline	Serum	NMR	mmol/L	0.047 (0.009)	0.055 (0.014)	0.054 (0.008)	0.059 (0.016)	-	0.191	0.664	0.062	**0.015**	**0.049**	0.201	0.560	0.737	0.013
	Lysophosphatidylcholine	Serum	NMR	mmol/L	0.261 (0.030)	0.316 (0.028)	0.240 (0.028)	0.317 (0.038)	-	0.257	0.803	0.047	**3.41E-08**	**3.51E-06**	0.682	0.197	0.461	0.061

Lipid metabolites – Ketone bodies[b]	3-Hydroxybutyrate	Plasma	NMR	mmol/L	0.064 [0.052 – 0.136]	0.078 [0.043 – 0.103]	0.056 [0.028 – 0.076]	0.061 [0.038 – 0.079]	Log	0.142	0.640	0.075	0.159	0.318	0.070	0.388	0.615	0.027
	Acetoacetate	Plasma	NMR	mmol/L	0.032 [0.022 – 0.071]	0.036 [0.025 – 0.048]	0.027 [0.017 – 0.035]	0.030 [0.021 – 0.042]	Log	0.143	0.640	0.075	0.241	0.435	0.049	0.540	0.722	0.014
	Acetone	Plasma	NMR	mmol/L	0.018 [0.015 – 0.022]	0.018 [0.015 – 0.023]	0.016 [0.016 – 0.020]	0.019 [0.015 – 0.023]	Log	0.610	0.897	0.009	0.310	0.508	0.037	0.348	0.615	0.032
Lipid metabolites – Acylcarnitines[a]	Carnitine (C:0)	Plasma	LC/MS	µmol/L	20.7 (4.7)	19.9 (4.5)	18.7 (5.0)	19.3 (4.7)	-	0.466	0.897	0.019	0.878	0.946	0.001	**0.039**	0.325	0.144
	Acetylcarnitine (C2:0)	Plasma	LC/MS	µmol/L	11.4 (3.0)	11.9 (2.5)	9.6 (2.4)	9.9 (1.9)	-	**0.030**	0.464	0.157	0.374	0.566	0.028	0.881	0.946	0.001
	Propionylcarnitine (C3:0)	Plasma	LC/MS	µmol/L	0.319 [0.257 – 0.382]	0.299 [0.249 – 0.382]	0.302 [0.228 – 0.344]	0.295 [0.250 – 0.357]	Log	0.357	0.855	0.030	0.906	0.962	0.001	0.380	0.615	0.028
	Butyrylcarnitine (C4:0)	Plasma	LC/MS	µmol/L	0.159 [0.137 – 0.222]	0.187 [0.139 – 0.219]	0.131 [0.114 – 0.166]	0.123 [0.109 – 0.168]	Log	**0.034**	0.464	0.151	0.882	0.946	0.001	0.574	0.739	0.011
	Isobutyrylcarnitine (C4:0-iso)	Plasma	LC/MS	µmol/L	0.093 [0.069 – 0.121]	0.079 [0.070 – 0.120]	0.087 [0.060 – 0.171]	0.099 [0.068 – 0.147]	Log	0.640	0.897	0.008	0.713	0.807	0.005	0.854	0.926	0.001
	Valerylcarnitine (C5)[†]	Plasma	LC/MS	µmol/L	0.076 [0.066 – 0.100]	0.077 [0.068 – 0.098]	0.076 [0.059 – 0.088]	0.073 [0.068 – 0.091]	Log	0.366	0.855	0.028	0.427	0.613	0.024	0.180	0.443	0.064
	Hydroxyisovalerylcarnitine (C5:0-OH)	Plasma	LC/MS	µmol/L	0.034 [0.030 – 0.049]	0.036 [0.030 – 0.047]	0.038 [0.032 – 0.043]	0.040 [0.032 – 0.042]	X	0.806*	0.897*	NA	0.187*	0.357*	NA	0.305*	0.615*	NA
	Glutarylcarnitine (C5:0-DC)	Plasma	LC/MS	µmol/L	0.069 [0.063 – 0.082]	0.067 [0.061 – 0.080]	0.079 [0.071 – 0.087]	0.081 [0.079 – 0.085]	Log	0.140	0.640	0.076	0.537	0.666	0.014	0.089	0.353	0.100
	Methylglutarylcarnitine (C5-M-DC)	Plasma	LC/MS	µmol/L	0.050 [0.037 – 0.057]	0.047 [0.038 – 0.056]	0.039 [0.035 – 0.055]	0.042 [0.036 – 0.063]	Inverse	0.922	0.931	0.000	0.066	0.155	0.115	**0.022**	0.325	0.173
	Methylcrotonylcarnitine (C5:1)	Plasma	LC/MS	µmol/L	0.010 [0.009 – 0.014]	0.011 [0.010 – 0.012]	0.011 [0.010 – 0.014]	0.013 [0.010 – 0.014]	X	0.174*	0.641*	NA	0.253*	0.449*	NA	0.775*	0.855*	NA
	Hexanoylcarnitine (C6:0)	Plasma	LC/MS	µmol/L	0.044 [0.038 – 0.049]	0.042 [0.039 – 0.065]	0.036 [0.034 – 0.044]	0.036 [0.031 – 0.039]	Inverse	**0.011**	0.376	0.210	0.861	0.943	0.001	0.086	0.353	0.101
	Octanoylcarnitine (C8:0)	Plasma	LC/MS	µmol/L	0.145 [0.119 – 0.208]	0.156 [0.131 – 0.254]	0.159 [0.131 – 0.176]	0.147 [0.126 – 0.165]	Inverse	0.402	0.855	0.025	0.532	0.666	0.014	**0.026**	0.325	0.165
	Octenoylcarnitine (C8:1)	Plasma	LC/MS	µmol/L	0.138 [0.101 – 0.159]	0.143 [0.125 – 0.170]	0.117 [0.085 – 0.138]	0.135 [0.101 – 0.155]	Log	0.241	0.775	0.049	**0.005**	**0.018**	0.251	0.941	0.982	0.000
	Decanoylcarnitine (C10:0)	Plasma	LC/MS	µmol/L	0.284 [0.216 – 0.425]	0.320 [0.261 – 0.565]	0.347 [0.255 – 0.398]	0.306 [0.238 – 0.371]	Inverse	0.745	0.897	0.004	0.478	0.648	0.018	**0.038**	0.325	0.145
	Decenoylcarnitine (C10:1)	Plasma	LC/MS	µmol/L	0.177 [0.162 – 0.193]	0.189 [0.178 – 0.281]	0.213 [0.178 – 0.262]	0.213 [0.160 – 0.228]	Inverse	0.822	0.897	0.002	0.748	0.829	0.004	0.065	0.346	0.117
	Dodecanoylcarnitine (C12:0)	Plasma	LC/MS	µmol/L	0.093 [0.073 – 0.128]	0.116 [0.088 – 0.160]	0.123 [0.094 – 0.166]	0.097 [0.087 – 0.172]	Log	0.808	0.897	0.002	0.514	0.655	0.015	0.081	0.352	0.105
	Hydroxydodecanoylcarnitine-a (C12:0-OH-a)	Plasma	LC/MS	µmol/L	0.012 [0.008 – 0.013]	0.015 [0.012 – 0.021]	0.019 [0.014 – 0.025]	0.016 [0.014 – 0.024]	Log	**0.036**	0.464	0.148	0.052	0.133	0.129	0.061	0.346	0.120
	Hydroxydodecanoylcarnitine-a (C12:0-OH-b)	Plasma	LC/MS	µmol/L	0.024 [0.015 – 0.027]	0.023 [0.018 – 0.027]	0.030 [0.022 – 0.035]	0.024 [0.021 – 0.030]	Log	0.168	0.640	0.067	0.393	0.570	0.026	0.648	0.783	0.008
	Dodecenylcarnitine (C12:1)	Plasma	LC/MS	µmol/L	0.127 [0.094 – 0.139]	0.135 [0.103 – 0.171]	0.154 [0.125 – 0.185]	0.134 [0.116 – 0.172]	Log	0.298	0.806	0.040	0.736	0.824	0.004	0.112	0.372	0.088
	Tetradecanoylcarnitine (C14:0)	Plasma	LC/MS	µmol/L	0.045 [0.039 – 0.058]	0.051 [0.041 – 0.059]	0.045 [0.037 – 0.057]	0.042 [0.037 – 0.056]	Log	0.407	0.855	0.025	0.961	0.972	8.90E-05	0.340	0.615	0.032
	Hydroxytetradecanoylcarnitine (C14:0-OH)	Plasma	LC/MS	µmol/L	0.024 [0.020 – 0.029]	0.027 [0.024 – 0.029]	0.028 [0.025 – 0.036]	0.029 [0.025 – 0.032]	Log	0.281	0.806	0.041	0.588	0.698	0.011	0.244	0.547	0.048
	Tetradecenoylcarnitine (C14:1)	Plasma	LC/MS	µmol/L	0.184 [0.130 – 0.200]	0.188 [0.155 – 0.248]	0.186 [0.158 – 0.251]	0.170 [0.152 – 0.267]	Log	0.828	0.897	0.002	0.435	0.613	0.022	0.137	0.409	0.077
	Tetradecadienylcarnitine (C14:2)	Plasma	LC/MS	µmol/L	0.064 [0.057 – 0.074]	0.080 [0.066 – 0.096]	0.086 [0.064 – 0.102]	0.075 [0.062 – 0.104]	Log	0.658	0.897	0.007	0.638	0.730	0.008	0.107	0.367	0.090
	Hexadecanoylcarnitine (C16:0)	Plasma	LC/MS	µmol/L	0.102 [0.087 – 0.111]	0.092 [0.088 – 0.107]	0.093 [0.087 – 0.099]	0.086 [0.088 – 0.091]	Log	0.088	0.640	0.101	0.084	0.183	0.103	0.944	0.982	1.7E-04
	Hydroxyhexadecanoylcarnitine (C16:0-OH)	Plasma	LC/MS	µmol/L	0.004 (0.002)	0.004 (0.001)	0.003 (0.001)	0.003 (0.001)	-	0.123	0.640	0.083	0.304	0.508	0.038	0.654	0.783	0.007
	Hexadecenylcarnitine (C16:1)	Plasma	LC/MS	µmol/L	0.036 [0.031 – 0.045]	0.036 [0.031 – 0.048]	0.038 [0.032 – 0.043]	0.037 [0.033 – 0.039]	Log	0.685	0.897	0.006	0.607	0.711	0.010	0.101	0.367	0.093
	Octadecanoylcarnitine (C18:0)	Plasma	LC/MS	µmol/L	0.058 [0.051 – 0.075]	0.058 [0.050 – 0.067]	0.057 [0.052 – 0.062]	0.061 [0.048 – 0.068]	Log	0.714	0.897	0.005	0.327	0.511	0.034	0.642	0.783	0.008
	Octadecenoylcarnitine (C18:1)	Plasma	LC/MS	µmol/L	0.112 (0.028)	0.112 (0.028)	0.114 (0.020)	0.109 (0.019)	-	0.907	0.931	4.98E-04	0.468	0.643	0.019	0.515	0.708	0.015

	Octadecadienylcarnitine (C18:2)	Plasma	LC/MS	µmol/L	0.037 [0.032 – 0.046]	0.042 [0.032 – 0.045]	0.039 [0.035 – 0.042]	0.038 [0.036 – 0.042]	Log	0.822	0.897	0.002	0.497	0.648	0.017	0.491	0.684	0.017
Lipid metabolites – Cholesterol metabolites[b]	Total cholesterol	Plasma	NMR	mmol/L	4.07 [3.82 – 4.61]	4.03 [3.72 – 4.24]	4.17 [3.88 – 4.56]	3.87 [3.48 – 4.35]	Log	0.899	0.931	0.001	**9.70E-05**	**0.001**	0.424	0.398	0.622	0.026
	VLDL cholesterol	Plasma	NMR	mmol/L	0.48 (0.08)	0.44 (0.07)	0.44 (0.13)	0.45 (0.15)	-	0.745	0.897	0.004	0.175	0.339	0.065	**0.010**	0.247	0.216
	LDL cholesterol	Plasma	NMR	mmol/L	1.57 (0.31)	1.50 (0.23)	1.55 (0.30)	1.40 (0.28)	-	0.567	0.897	0.012	**0.002**	**0.010**	0.292	0.229	0.524	0.051
	HDL cholesterol	Plasma	NMR	mmol/L	1.55 [1.35 – 1.61]	1.45 [1.32 – 1.57]	1.47 [1.41 – 1.68]	1.40 [1.30 – 1.58]	Log	0.626	0.897	0.009	**1.06E-04**	**0.001**	0.421	0.169	0.443	0.066
	Total esterified cholesterol	Plasma	NMR	mmol/L	3.04 [2.80 – 3.42]	2.99 [2.76 – 3.14]	3.12 [2.91 – 3.36]	2.83 [2.57 – 3.23]	Log	0.908	0.931	4.80E-05	**9.50E-05**	**0.001**	0.425	0.298	0.613	0.039
	Total free cholesterol	Plasma	NMR	mmol/L	1.05 [1.01 – 1.18]	1.04 [0.95 – 1.09]	1.10 [0.98 – 1.19]	1.01 [0.91 – 1.13]	Log	0.894	0.931	0.001	**1.49E-04**	**0.001**	0.407	0.980	0.989	0.000
Lipid metabolites – Apolipoproteins[b]	Apolipoprotein B	Plasma	NMR	mmol/L	0.706 (0.106)	0.676 (0.086)	0.694 (0.114)	0.664 (0.115)	-	0.763	0.897	0.003	**0.001**	**0.008**	0.317	0.989	0.989	$7.00x10^{-6}$
	Apolipoprotein A1	Plasma	NMR	mmol/L	1.53 (0.19)	1.48 (0.16)	1.58 (0.19)	1.50 (0.19)	-	0.615	0.897	0.009	**1.33E-04**	**0.001**	0.412	0.384	0.615	0.027
Lipid metabolites – Lipoproteins[b, $]	Total lipoprotein particles	Plasma	NMR	µmol/L	18.1 [15.5 – 18.9]	17.2 [15.4 – 18.6]	18.3 [16.3 – 18.7]	16.1 [15.4 – 18.2]	X	0.595*	0.897*	NA	**0.002***	**0.010***	NA	0.187*	0.443*	NA
	VLDL particles	Plasma	NMR	µmol/L	0.108 [0.100 – 0.116]	0.098 [0.089 – 0.106]	0.102 [0.076 – 0.116]	0.101 [0.077 – 0.114]	Log	0.610	0.897	0.009	**0.048**	0.126	0.133	0.065	0.346	0.116
	LDL particles	Plasma	NMR	µmol/L	1.02 (0.17)	0.99 (0.14)	1.01 (0.17)	0.95 (0.17)	-	0.825	0.897	0.002	**4.20E-04**	**0.003**	0.364	0.326	0.615	0.034
	HDL particles	Plasma	NMR	µmol/L	16.8 [14.2 – 17.7]	15.9 [14.1 – 17.1]	16.9 [14.7 – 17.3]	14.8 [14.3 – 16.8]	X	0.567*	0.897*	NA	**0.003***	**0.013***	NA	0.148*	0.412*	NA
	Chylomicrons & XXL-VLDL particles	Plasma	NMR	nmol/L	0.18 [0.03 – 0.60]	0.09 [0.03 – 0.43]	0.02 [0.02 – 0.23]	0.35 [0.04 – 0.62]	X	**0.045***	0.464*	NA	0.229*	0.429	NA	**0.007***	0.247*	NA
	XL-VLDL particles	Plasma	NMR	nmol/L	1.85 [1.56 – 2.39]	1.58 [1.26 – 2.04]	1.68 [1.19 – 1.85]	1.64 [1.21 – 1.99]	X	0.174*	0.640*	NA	0.491*	0.648*	NA	**0.041***	0.325*	NA
	L-VLDL particles	Plasma	NMR	nmol/L	6.79 [6.21 – 8.06]	6.03 [5.05 – 7.57]	6.32 [5.02 – 7.13]	5.94 [4.60 – 6.98]	Log	0.312	0.812	0.037	0.083	0.183	0.104	0.103	0.367	0.092
	M-VLDL particles	Plasma	NMR	nmol/L	25.3 [23.4 – 29.7]	24.5 [21.2 – 27.2]	26.1 [17.8 – 28.4]	23.0 [17.7 – 27.2]	Log	0.403	0.855	0.025	**0.016**	**0.051**	0.191	0.363	0.615	0.030
	S-VLDL particles	Plasma	NMR	nmol/L	30.5 [27.9 – 34.1]	28.9 [24.9 – 33.3]	32.1 [20.0 – 34.8]	27.7 [20.3 – 32.7]	Log	0.516	0.897	0.015	0.125	0.257	0.082	0.347	0.615	0.032
	XS-VLDL particles	Plasma	NMR	nmol/L	40.0 [35.6 – 43.7]	37.0 [34.0 – 39.9]	38.2 [33.7– 45.1]	39.7 [34.1 – 44.3]	Log	0.795	0.897	0.002	0.316	0.508	0.036	**0.014**	0.294	0.196
	IDL particles	Plasma	NMR	nmol/L	246 (36)	231 (28)	247 (32)	242 (36)	-	0.678	0.897	0.006	**0.002**	**0.010**	0.297	0.079	0.352	0.106
	L-LDL particles	Plasma	NMR	nmol/L	617 (87)	598 (85)	601 (99)	591 (105)	-	0.739	0.897	0.004	**0.042**	0.114	0.140	0.482	0.680	0.018
	M-LDL particles	Plasma	NMR	nmol/L	254 (57)	244 (40)	255 (60)	222 (48)	-	0.574	0.897	0.011	**0.004**	**0.015**	0.265	0.093	0.354	0.098
	S-LDL particles	Plasma	NMR	nmol/L	152 (28)	145 (21)	149 (22)	138 (20)	-	0.544	0.897	0.013	**0.001**	**0.008**	0.315	0.410	0.631	0.024
	XL-HDL particles	Plasma	NMR	nmol/L	240 [205 – 261]	224 [193 – 270]	216 [183 – 283]	236 [191 – 279]	Log	0.560	0.897	0.012	0.497	0.648	0.017	0.267	0.574	0.044
	L-HDL particles	Plasma	NMR	µmol/L	1.78 [1.53 – 1.98]	1.70 [1.35 – 1.83]	1.61 [1.32 – 2.18]	1.63 [1.30 – 1.98]	Log	0.558	0.897	0.012	0.066	0.155	0.116	0.526	0.713	0.014
	M-HDL particles	Plasma	NMR	µmol/L	4.21 (0.73)	4.02 (0.62)	4.37 (0.71)	4.09 (0.67)	-	0.644	0.897	0.008	**1.41E-04**	**0.001**	0.409	0.380	0.615	0.028
	S-HDL particles	Plasma	NMR	µmol/L	10.03 (1.29)	9.81 (0.99)	10.11 (1.19)	9.38 (0.965)	-	0.644	0.897	0.008	**0.016**	**0.051**	0.189	0.181	0.443	0.063
Other metabolites – Fluid balance[b]	Creatinine	Plasma	NMR	µmol/L	60.6 [59.1 – 66.1]	59.8 [55.4 – 66.9]	63.0 [61.1 – 69.6]	67.9 [64.1 – 71.1]	Log	0.078	0.640	0.107	0.382	0.570	0.027	**0.035**	0.325	0.149
	Albumin	Plasma	NMR	g/L	38.2 (2.6)	38.3 (2.1)	39.1 (2.8)	38.9 (1.9)	-	0.370	0.855	0.029	0.951	0.972	1.36E-04	0.623	0.773	0.009

Normally distributed data is represented as mean (SD) and non-normally distributed data is represented as mean [IQR]. Raw P-values were adjusted using an FDR of 10%. Significant raw P-values are indicated in **bold**. Significant FDR-adjusted P-values (< 0.10) are indicated in **underlined bold**.

[#]Transformations (log, inverse, inverse square root) were applied on not-normally distributed variables. Variables that did not achieve a normal distribution after transformation are represented with an X. For these variables non-parametric tests were used: the fitness level effect (P_{group}) was analyzed using a Mann-Whitney U test on the ranked baseline values; the exercise effect ($P_{exercise}$) was analyzed using a Wilcoxon-Signed rank test on the ranked baseline and post-exercise values; the interaction effect ($P_{group*exercise}$) was analyzed using a Mann-Whitney U test on the ranked difference between baseline and post-exercise values. These p-values are marked with an asterisk (*). NA = not applicable

[$]Abbreviations: FA = fatty acid, PUFA = polyunsaturated fatty acid, ARA & EPA = arachidonic acid & eicosapentaenoic acid, DHA = docosahexaenoic acid, MUFA = monounsaturated fatty acid, SFA = saturated fatty acidd, XXL = extremely large, XL = very large, L = large, M = medium, I = intermediate, S = small, XS = very small.

[†]Refer to the two isomers 2-methylburytylcarnitine (C4:0-2M) and isovalerylcarnitine (C5:0).

[a] Analyzed using the EURECAT platform.

[b] Analyzed using the Nightingale platform.
