# Supplementary material for: Single and Joined Behaviour of Circulating Biomarkers and Metabolic Parameters in High-Fit and Low-Fit Healthy Females
Source: Int J Mol Sci. 2023 Feb 20;24(4):4202. doi: 10.3390/ijms24044202 (PMC9960642; doi:10.3390/ijms24044202)
Supplement: Supplementary file 1 [file ijms-24-04202-s001.zip › Tables S5,6,7.pdf]

Supplementary Table S5: Main effect analysis of peptide hormone markers

**Biomarker**	**Fitness level effect**				**Recent exercise effect**			
	**RM-ANOVA**		**Main effect analysis**		**RM-ANOVA**		**Main effect analysis**	
	**P_{group} raw**	**P_{group} adj**	**P_{group} raw**	**P_{group} adj**	**$P_{exercise}$ raw**	**$P_{exercise}$ adj**	**$P_{exercise}$ raw**	**$P_{exercise}$ adj**
Leptin	**0.001**	<u>**0.076**</u>	**0.001**	<u>**0.076**</u>	0.391	0.570	0.411	0.605
Insulin	**0.039**	0.464	**0.039**	0.464	**0.037**	0.105	**0.035**	0.101
Adiponectin	0.914	0.931	0.914	0.931	**1.55E-04**	<u>**0.001**</u>	1.88E-04	<u>**0.002**</u>

Raw P-values were adjusted using an FDR of 10%. Significant raw P-values are indicated in **bold**. Significant FDR-adjusted P-values (< 0.10) are indicated in <u>**underlined bold**</u>.

Supplementary Table S6: Main effect analysis of inflammation and oxidative stress related markers

**Biomarker**	**Fitness level effect**				**Recent exercise effect**			
	**RM-ANOVA**		**Main effect analysis**		**RM-ANOVA**		**Main effect analysis**	
	**P_{group} raw**	**P_{group} adj**	**P_{group} raw**	**P_{group} adj**	**$P_{exercise}$ raw**	**$P_{exercise}$ adj**	**$P_{exercise}$ raw**	**$P_{exercise}$ adj**
TNF-a	0.693	0.897	0.693	0.870	**1.20E-05**	<u>**3.09E-04**</u>	**4.70E-05**	<u>**0.001**</u>
IL-6	0.154	0.640	0.154	0.640	**0.004**	<u>**0.017**</u>	**0.007**	<u>**0.025**</u>
IL-10	**0.025**	0.464	**0.025**	0.464	**3.46E-04**	<u>**0.003**</u>	**4.44E-04**	<u>**0.003**</u>
CRP	0.065	0.606	0.065	0.606	**3.31E-04**	<u>**0.003**</u>	**3.38E-04**	<u>**0.003**</u>
Soluble CD14	0.546	0.897	0.546	0.870	**0.004**	<u>**0.015**</u>	**0.005**	<u>**0.018**</u>
MCP-1	0.379	0.855	0.379	0.806	**1.21E-07**	<u>**4.16E-06**</u>	**9.03E-06**	<u>**2.32E-04**</u>
Soluble ICAM-1	0.160	0.640	0.160	0.640	0.939	0.972	0.938	0.979
LBP	0.604	0.897	0.604	0.870	0.620	0.718	0.614	0.716
N-acetyl glycoproteins	0.513	0.897	0.513	0.870	**1.02E-07**	<u>**4.16E-06**</u>	**6.03E-07**	<u>**3.11E-05**</u>
Oxidized LDL	0.679	0.897	0.679	0.870	0.315	0.508	0.309	0.514

Raw P-values were adjusted using an FDR of 10%. Significant raw P-values are indicated in **bold**. Significant FDR-adjusted P-values (< 0.10) are indicated in <u>**underlined bold**</u>.

Supplementary Table S7: Main effect analysis of metabolic markers

**Metabolism class**	**Biomarker**	**Fitness level effect**				**Recent exercise effect**			
		**RM-ANOVA**		**Main effect analysis**		**RM-ANOVA**		**Main effect analysis**	
		**P_{group} raw**	**P_{group} adj**	**P_{group} raw**	**P_{group} adj**	**$P_{exercise}$ raw**	**$P_{exercise}$ adj**	**$P_{exercise}$ raw**	**$P_{exercise}$ adj**
Protein and amino acid related metabolites	Isoleucine	0.287	0.806	0.287	0.784	0.447	0.622	0.428	0.614
	Leucine	0.081	0.640	0.081	0.640	0.298	0.508	0.286	0.499
	Valine	0.991	0.931	0.991	0.991	**0.002**	**0.009**	**0.001**	**0.008**
	Alanine	0.187	0.664	0.187	0.655	0.060	0.146	0.055	0.144
	Phenylalanine	0.129	0.640	0.129	0.640	0.927	0.972	0.983	0.992
	Glutamine	0.406	0.855	0.406	0.806	**0.005**	**0.018**	**0.004**	**0.017**
	Glutamate	0.315	0.812	0.315	0.792	0.984	0.972	0.947	0.979
	Glycine	0.148	0.640	0.148	0.640	0.566	0.685	0.543	0.674
	Methionine	**0.010**	0.376	**0.010**	0.376	0.963	0.972	0.999	0.999
	Tyrosine	0.676	0.897	0.676	0.870	0.549	0.673	0.526	0.665
	Tryptophan	0.592	0.897	0.592	0.870	0.270	0.472	0.268	0.477
	Betaine	0.740	0.897	0.740	0.882	**0.002**	**0.011**	**0.002**	**0.010**
Carbohydrate and TCA cycle metabolites	Glucose	0.620	0.897	0.620	0.870	0.363	0.559	0.356	0.560
	Lactate	0.382	0.855	0.382	0.806	0.589	0.698	0.583	0.698
	Pyruvate	0.531	0.897	0.531	0.870	0.123	0.257	0.122	0.255
	Citrate	0.204	0.677	0.204	0.656	0.238	0.435	0.247	0.455
Lipid metabolites – Fatty acids	Total FA	0.310	0.812	0.310	0.792	**0.040**	0.111	0.036	0.101
	PUFA	0.127	0.640	0.127	0.640	0.074	0.169	0.100	0.218
	Omega-3 FA	0.197	0.677	0.197	0.655	0.515	0.655	0.527	0.665
	ARA & EPA	0.158	0.640	0.158	0.640	0.097	0.208	0.116	0.250
	DHA	**0.041**	0.464	**0.041**	0.464	**0.032**	**0.095**	**0.029**	**0.088**
	Linoleic acid	0.165	0.640	0.165	0.640	**0.012**	**0.041**	**0.021**	**0.065**

	MUFA	0.276	0.806	0.276	0.784	**0.023**	**0.070**	**0.036**	0.101
	Oleic acid	0.426	0.878	0.426	0.828	0.053	0.134	0.070	0.169
	SFA	0.351	0.855	0.351	0.806	**0.012**	**0.041**	**0.010**	**0.037**
	Phosphoglycerides	0.814	0.897	0.814	0.888	**2.24E-04**	**0.002**	**1.87E-04**	**0.002**
Lipid metabolites – Cholines	Choline	0.191	0.897	0.191	0.655	**0.015**	**0.049**	**0.014**	**0.049**
	Lysophosphatidylcholine	0.257	0.803	0.257	0.780	**3.41E-08**	**3.51E-06**	**3.04E-07**	**3.11E-05**
	3-Hydroxybutyrate	0.142	0.640	0.142	0.640	0.159	0.318	0.157	0.316
Lipid metabolites – Ketone bodies	Acetate	0.166	0.640	0.166	0.640	0.160	0.318	0.161	0.319
	Acetoacetate	0.143	0.640	0.143	0.640	0.241	0.435	0.235	0.441
	Acetone	0.610	0.897	0.610	0.870	0.310	0.508	0.309	0.514
	Carnitine (C:0)	0.466	0.897	0.466	0.870	0.878	0.946	0.885	0.950
	Acetylcarnitine (C2:0)	**0.030**	0.464	**0.030**	0.464	0.374	0.566	0.366	0.562
	Propionylcarnitine (C3:0)	0.357	0.855	0.357	0.806	0.906	0.962	0.906	0.962
	Butyrylcarnitine (C4:0)	**0.034**	0.464	**0.034**	0.464	0.882	0.946	0.881	0.950
	Isobutyrylcarnitine (C4:0-iso)	0.640	0.897	0.640	0.870	0.713	0.807	0.708	0.802
	2-Methylbutyrylcarnitine (C4:0-2M)	0.367	0.855	0.367	0.806	0.429	0.613	0.436	0.614
	Isovalerylcarnitine (C5:0)	0.366	0.855	0.366	0.806	0.427	0.613	0.436	0.614
	Hydroxyisovalerylcarnitine (C5:0-OH)	0.806*	0.897*	0.806*	0.888*	0.187*	0.357*	0.187*	0.376*
Lipid metabolites – Acylcarnitines	Glutarylcarnitine (C5:0-DC)	0.140	0.640	0.140	0.640	0.537	0.666	0.550	0.675
	Methylglutarylcarnitine (C5-M-DC)	0.922	0.931	0.922	0.931	0.066	0.155	0.087	0.200
	Methylcrotonylcarnitine (C5:1)	0.174*	0.641*	0.174*	0.640*	0.253*	0.449*	0.253*	0.457*
	Hexanoylcarnitine (C6:0)	**0.011**	0.376	**0.011**	0.376	0.861	0.943	0.866	0.949
	Octanoylcarnitine (C8:0)	0.402	0.855	0.402	0.806	0.532	0.666	0.561	0.680
	Octenoylcarnitine (C8:1)	0.241	0.775	0.241	0.752	**0.005**	**0.018**	**0.004**	**0.017**
	Decanoylcarnitine (C10:0)	0.745	0.897	0.745	0.882	0.478	0.648	0.504	0.657
	Decenoylcarnitine (C10:1)	0.822	0.897	0.822	0.888	0.748	0.829	0.759	0.840
	Dodecanoylcarnitine (C12:0)	0.808	0.897	0.808	0.888	0.514	0.655	0.529	0.665
	Hydroxydodecanoylcarnitine-a (C12:0-OH-a)	**0.036**	0.464	**0.036**	0.464	0.052	0.133	0.062	0.155

	Hydroxydodecanoylcarnitine-a (C12:0-OH-b)	0.168	0.640	0.168	0.640	0.393	0.570	0.386	0.585
	Dodecenylcarnitine (C12:1)	0.298	0.806	0.289	0.784	0.736	0.824	0.743	0.832
	Tetradecanoylcarnitine (C14:0)	0.407	0.855	0.407	0.806	0.961	0.972	0.961	0.980
	Hydroxytetradecanoylcarnitine (C14:0-OH)	0.281	0.806	0.281	0.784	0.588	0.698	0.590	0.699
	Tetradecenoylcarnitine (C14:1)	0.828	0.897	0.828	0.888	0.435	0.613	0.445	0.619
	Tetradecadienylcarnitine (C14:2)	0.658	0.897	0.658	0.870	0.638	0.730	0.648	0.741
	Hexadecanoylcarnitine (C16:0)	0.088	0.640	0.088	0.640	0.084	0.183	0.078	0.183
	Hydroxyhexadecanoylcarnitine (C16:0-OH)	0.123	0.640	0.123	0.640	0.304	0.508	0.297	0.509
	Hexadecenylcarnitine (C16:1)	0.685	0.897	0.685	0.870	0.607	0.711	0.618	0.716
	Octadecanoylcarnitine (C18:0)	0.714	0.897	0.714	0.882	0.327	0.511	0.321	0.517
	Octadecenoylcarnitine (C18:1)	0.907	0.931	0.907	0.931	0.468	0.643	0.464	0.637
	Octadecadienylcarnitine (C18:2)	0.822	0.897	0.822	0.888	0.497	0.648	0.493	0.657
Lipid metabolites – Cholesterol metabolites	Total cholesterol	0.899	0.931	0.905	0.931	**9.70E-05**	**0.001**	**8.70E-05**	**0.001**
	VLDL cholesterol	0.745	0.897	0.745	0.882	0.175	0.339	0.220	0.427
	LDL cholesterol	0.567	0.897	0.567	0.870	**0.002**	**0.010**	**0.002**	**0.011**
	HDL cholesterol	0.626	0.897	0.627	0.870	**1.06E-04**	**0.001**	**1.19E-04**	**0.001**
	Total esterified cholesterol	0.908	0.931	0.908	0.931	**9.50E-05**	**0.001**	**1.19E-04**	**0.001**
	Total free cholesterol	0.894	0.931	0.897	0.931	**1.49E-04**	**0.001**	**1.07E-04**	**0.001**
Lipid metabolites – Apolipoproteins	Apolipoprotein B	0.763	0.897	0.763	0.888	**0.001**	**0.008**	**0.001**	**0.007**
	Apolipoprotein A1	0.615	0.897	0.616	0.870	**1.33E-04**	**0.001**	**1.19E-04**	**0.001**
Lipid metabolites – Lipoproteins	Total lipoprotein particles	0.595*	0.897*	0.595	0.870	**0.002***	**0.010***	**0.002**	**0.010**
	VLDL particles	0.610	0.897	0.609	0.870	**0.048**	0.126	0.057	0.146
	LDL particles	0.825	0.897	0.649	0.870	**4.20E-04**	**0.003**	**0.002**	**0.010**
	HDL particles	0.567*	0.897*	0.567*	0.870*	**0.003***	**0.013***	**0.003***	**0.013***
	Chylomicrons & XXL-VLDL particles	**0.045***	0.464*	**0.045***	0.464*	0.229*	0.429*	0.229*	0.437*
	XL-VLDL particles	0.174*	0.640*	0.174*	0.640*	0.491*	0.648*	0.491*	0.657*
	L-VLDL particles	0.312	0.812	0.312	0.792	0.083	0.183	0.092	0.205
	M-VLDL particles	0.403	0.855	0.403	0.806	**0.016**	**0.051**	**0.015**	**0.051**

	S-VLDL particles	0.516	0.897	0.516	0.870	0.125	0.257	0.124	0.255
	XS-VLDL particles	0.795	0.897	0.795	0.888	0.316	0.508	0.359	0.560
	IDL particles	0.678	0.897	0.659	0.870	**0.002**	**<u>0.010</u>**	**0.002**	**<u>0.011</u>**
	L-LDL particles	0.739	0.897	0.739	0.882	**0.042**	0.114	**0.040**	0.108
	M-LDL particles	0.574	0.897	0.574	0.870	**0.004**	**<u>0.015</u>**	**0.005**	**<u>0.018</u>**
	S-LDL particles	0.544	0.897	0.544	0.870	**0.001**	**<u>0.008</u>**	**0.001**	**<u>0.007</u>**
	XL-HDL particles	0.560	0.897	0.560	0.870	0.497	0.648	0.499	0.657
	L-HDL particles	0.558	0.897	0.558	0.870	0.066	0.155	0.063	0.155
	M-HDL particles	0.644	0.897	0.644	0.870	**1.41E-04**	**<u>0.001</u>**	**1.00E-06**	**<u>3.43E-05</u>**
	S-HDL particles	0.644	0.897	0.644	0.870	**0.016**	**<u>0.051</u>**	**0.018**	**<u>0.057</u>**
Other metabolites – Fluid balance[b]	Creatinine	0.078	0.640	0.078	0.640	0.382	0.570	0.411	0.605
	Albumin	0.370	0.897	0.370	0.806	0.951	0.972	0.951	0.979

Raw P-values were adjusted using an FDR of 10%. Significant raw P-values are indicated in **bold**. Significant FDR-adjusted P-values (< 0.10) are indicated in **<u>underlined bold</u>**. Biomarkers indicated with an asterisk (*) were analyzed non-parametrically.
